# Surgical Results of Patients with Peritoneal Carcinomatosis Treated with Cytoreductive Surgery Using a New Technique Named Aqua Dissection

**DOI:** 10.1155/2012/521487

**Published:** 2012-05-15

**Authors:** Y. Yonemura, A. Elnemr, Y. Endou, H. Ishibashi, A. Mizumoto, M. Miura, Yan Li

**Affiliations:** ^1^NPO Organization to Support Peritoneal Surface Malignancy Treatment, 1-26, Haruki-Moto-Machi, Kishiwada, Osaka, Japan; ^2^Department of Surgery, Kusatsu General Hospital, Shiga, Japan; ^3^Department of Surgery, Peritoneal Surface Malignancy Center, Kishiwada Tokushukai Hospital, Kishiwada, Japan; ^4^Department of Experimental Therapeutics, Cancer Research Institute, Kanazawa University, Kanazawa, Japan; ^5^Department of Anatomy, School of Medicine, Oita University, Oita, Japan; ^6^Department of Oncology, Zhongnam Hospital and Cancer Center, Wuhan University, Wuhan, China

## Abstract

During 2004 to 2011, 81, 420, and 166 patients with colorectal cancer (CRC), epithelial appendiceal neoplasm (APN), and gastric cancer (GC) with PC were treated with cytoreductive surgery (CRS) plus perioperative chemotherapy. CRS was performed by peritonectomy techniques using an aqua dissection. *Results*. Complete cytoreduction was done in 62/81 (76.5%), 228/420 (54.3%), and 101/166 (60.8%) of patients with CRC, APN, and GC. The main reasons of incomplete resections were involvement of all peritoneal regions and diffuse involvement of small bowel. The incidence (64%, 302/470) of CC-0 resection after introduction of an aqua dissection was significantly higher than before (42%, 82/197). A total of 41 (6.1%) patients died postoperatively. Major complication (grade 3-4 complications) occurred in 126 patients (18.9%). A reoperation was necessary in 36 patients (5.4%). By the multivariate analysis, PCI scores capable of serving as thresholds for favorable versus poor prognosis in each group and CC scores demonstrated as the independent prognostic factors. *Conclusions*. Peritonectomy using an aqua dissection improves the incidence of complete cytoreduction, and improves the survival of patients with PC. Patients with PCI larger than the threshold values should be treated with chemotherapy to improve the incidences of complete cytoreduction.

## 1. Introduction

The current state-of-the-art treatment for the peritoneal carcinomatosis (PC) from colorectal, appendiceal, and gastric cancers consists of a comprehensive management strategy using cytoreductive surgery (CRS) and perioperative intraperitoneal chemotherapy (PIC) [1–5]. Patients with a low tumor volume, well/moderately differentiated tumors, and complete cytoreduction may potentially benefit from combined treatment. No survival benefit has been reported by cytoreduction alone [3]. In contrast, CRS plus hyperthermic intraoperative intraperitoneal chemotherapy (HIPEC) confers a prolonged survival period [2, 3]. Among several prognostic factors, complete cytoreduction is the most important prognostic factor for a good outcome [1–3].

However, complete cytoreduction is sometimes difficult in patients with deep invasion into the liver hilum, lesser omentum, pelvic structures, liver parenchyma, or diffuse involvement of the mesentery and serosa of small bowel. Even by the most experienced surgeons in the world, the incidences of complete cytoreduction are reported 77% (617/802) [4]. However, the complete cytoreduction rate depends on the selection criteria for the CRS and the ability and experiences of the surgeons. In the present paper, our surgical techniques for the complete yet safe cytoreduction and the results after CRS will be reported; 81 (42.9%), 420 (72.7%) and 166 (51.5%).

## 2. Patients and Methods

### 2.1. Patients

Between June, 2004, and January, 2011, a total of 667 patients underwent CRS combined with PIC for peritoneal carcinomatosis from colorectal origin (*N* = 81), epithelial appendiceal neoplasm (*N* = 420), and gastric cancer (*N* = 166), led by a single surgeon (Y. Yonemura) at Kishiwada Tokushukai and Kusatsu General Hospital, Japan. The included patients were >19 and, <87 years old, with good performance status (World Health Organization Performance Status ≤2). All patients underwent extensive preoperative investigations, which included physical examination and abdominal, pelvic, and chest computed tomography (CT) scans to assess the extent of the disease involved. CT scans were performed following the administration of oral and intravenous contrast media. Signed informed consent was obtained from all patients.

### 2.2. Quantitative Evaluation of the Volume of PC and Assessment Completeness of Cytoreduction

Preoperatively, the tumor volume was quantified according to computed tomography (CT) scans using the Peritoneal Cancer Index (PCI, Washington Cancer Institute) [6, 7]. The abdomen and pelvis were divided into nine regions and the small bowel into four: each assigned a lesion size (LS) score of 0–3, representative of the largest implant visualized. LS-0 denotes the absence of implants, LS-1 indicates implants <0.25 cm, LS-2 implants between 0.25 and 5 cm, and LS-3 implants >5 cm or a confluence of disease. These figures amount to a final numerical score of 0–39.

### 2.3. Selection Criteria for CRS

CRS consists of numerous surgical procedures depending on the extent of peritoneal tumor manifestation. Surgery may include parietal and visceral peritonectomy, greater and lesser omentectomy, splenectomy, cholecystectomy, resection of the liver capsule, small bowel resections, colonic and rectal resections, gastrectomy, pancreatic resection, hysterectomy, ovariectomy, and urine bladder resection [8].

Patients who had the following criteria are excluded as candidates for peritonectomy: (1) evidence of lymph node involvement and distant hematogenous metastasis confirmed by computed tomography (CT), magnetic resonance imaging (MRI), or ^18^Fluorodeoxyglucose positron emission tomography (PET/CT), (2) progressive disease after preoperative chemotherapy, and (3) severe comorbidities or poor general condition.

### 2.4. Methods of CRS Using Peritonectomy Techniques

#### 2.4.1. Dissection Techniques of CRS

Under general anesthesia, midline incision was made from the xiphoid to the pubis, and PCI score was calculated in each case [8, 9].

For the tissue dissection, electrosurgical techniques are used. In electrosurgery, a generator delivers high frequency current greater than 200 kHz under high power electricity (100 Watt), and the tissue impedance converts electric current into thermal energy, resulting in the localized tissue heating and coagulation. We use the electrosurgical generator (Valleylab Inc., Boulder, CO, USA), on pure cut and adjusted to the maximum electrical power. The mainly used handpiece is the ball-tipped type. The 2 mm ball-tip electrode is used for dissecting on visceral surfaces, including stomach, small bowel, and colon. When more rapid tumor destruction is required, the 5 mm ball-tip can be used.

Before the tissue dissection with electrosurgery, a 5% dextran solution plus adrenaline (a concentration of 10^−6^) is injected into the dissection plane to separate the layers properly and to decrease bleeding. The technique is named the *aqua dissection* method and was started in January, 2008. The ball-tipped instrument is placed at the interface of tumor and normal tissues. The focal point for further dissection is placed on strong traction.

#### 2.4.2. Peritonectomy for Parietal Peritoneum

The skin incision deepened through the *linea alba* till reaching the extraperitoneal fat layer without opening the peritoneum. Then, both sides of the parietal peritoneum are peeled off from the posterior rectus sheath by the traction of the skin using stay silk sutures and anchoring the edge of the skin to the ring frame of the Munster retractor. As the plane between the posterior rectus sheath and peritoneum is loose in the area inferior to the arcuate line, the dissection is started in the lower parietal peritoneum. Then, the dissection between the peritoneum and the transversalis fascia is continued to the retroperitoneal space. The dissection continues deeply and in a counterclockwise direction, starting in the right flank till reaching the peritoneum covering the left cupula of the diaphragm. Then, the dissection is completed in the upper right side till reaching the anterior renal fascia, inferior vena cava, and posterior wall of the duodenum.

The peritoneum of the Morrison pouch and paracolic gutters on both sides is completely freed from retroperitoneum and is removed with the anterior parietal peritoneum. The ureters and gonadal vessels are identified and taped. In males, the gonadal vessels should be preserved but are removed with the ovary in females.

The dissected parietal peritoneum is opened in the midline, and extensive wash and aspiration of the peritoneal cavity ten times using one liter of normal saline each time is done. The purpose of the washing is to remove peritoneal free cancer cells and mucinous materials from the peritoneal cavity. During the washing surgeons decide the operation plan.

#### 2.4.3. Peritonectomy of the Undersurface of Diaphragm

If the undersurface of the diaphragm is involved, stripping of peritoneum from the right and left hemidiaphragm is done. The falciform and round ligament are taken down and resected completely. Bleeding from diaphragmatic muscle is stopped by argon beam coagulation (ABC) which has a penetration depth of coagulation limited to a few millimeters. Advantages of ABC include the ability to coagulate broad surface areas and larger vessels.

**Figure 1:**
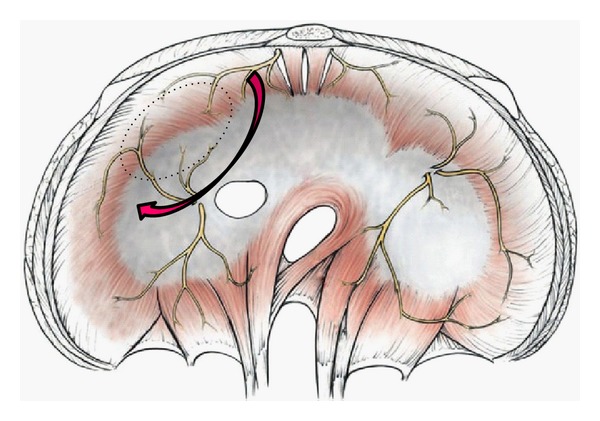
Cancer cells tend to invade the muscle layer of the encircled area of the right hemidiaphragm, where is the boundary of bare area and peritoneal reflection. Bare area below the invaded.

**Figure 2:**
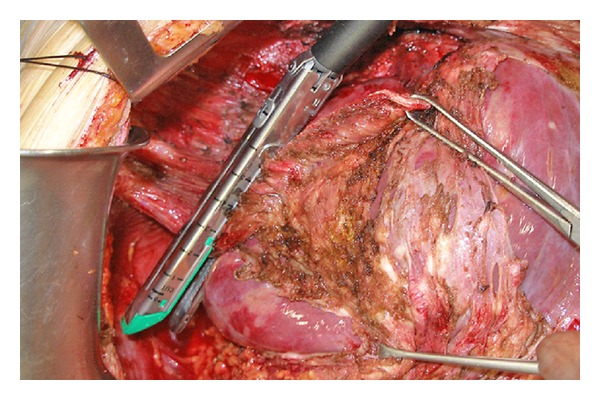
Partial resection of the diaphragm by using a linear stapler.

Figure 1 shows the area where cancer cells tend to invade into the muscle layer of the right hemidiaphragm. After the blunt dissection of the posterior space of the invaded diaphragm with finger between diaphragm and the bare area, partial resection of the full thickness of the right diaphragmatic cupula infiltrated by tumor is excised using a linear stapler (Figure 2). The staple line is then reinforced with an absorbable suture material.

#### 2.4.4. Perigastric Peritonectomy

**Figure 3:**
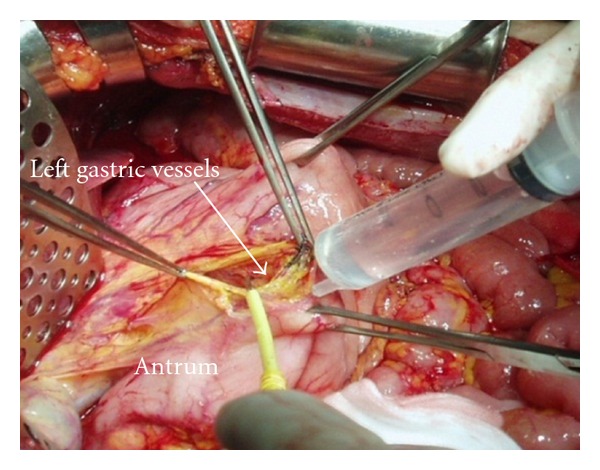
A 5% dextrose solution is injected in the incision site on the lesser curvature, and the left gastric vessels are identified and taped. Aqua dissection technique.

**Figure 4:**
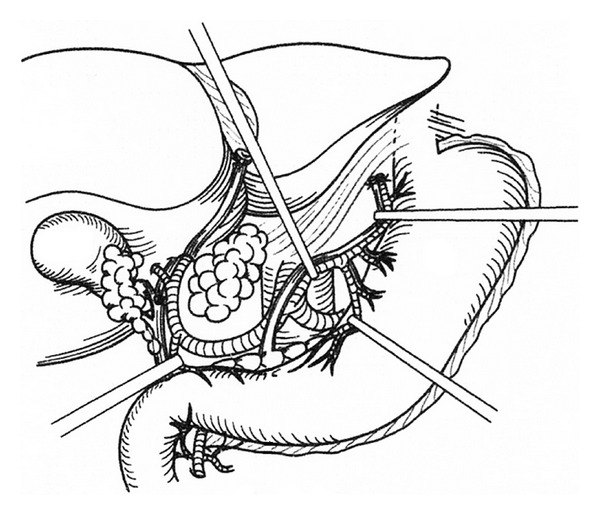
Preservation of the left gastric vessels and whole stomach. Surgical techniques of the removal of lesser omental tumors.

A greater omentectomy is performed with combination of splenectomy and the resection of anterior leaf of mesocolon. If the omentum is free of disease, gastroepiploic arcade is preserved after taping the root of the vessels. Greater omentum is removed with the right gastroepiploic vessels if it is involved with bulky tumor. Splenic artery and vein are identified and ligated at the splenic hilum. If the right gastroepiploic vessels and spleen are removed, left gastric artery and vein should be preserved. After the left lobe of the liver is freed from the left triangular ligament, resection of the lesser omentum along the Arantius duct is started. Next the small incisions are made on the peritoneal attachment to the stomach wall, and the 5% dextran solution is injected through the incision (*aqua dissection* technique), resulting in the separation of lesser omental tumor from the left gastric vessels. In the appendiceal tumor and colorectal cancer, the boundary between tumor and normal tissue is clear, and the omental tumors can be easily removed by the traction of the taped vessels (Figures 3 and 4). The whole stomach is preserved by the preservation of the left gastric vessels without perforation.

**Figure 5:**
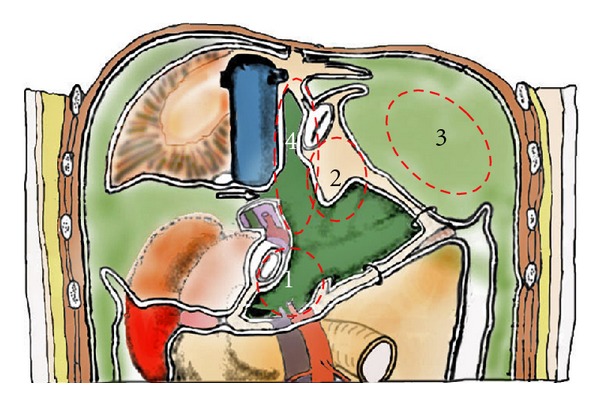
The parts of gastric wall liable to involvement by the disease process (dotted line): (1) the posterior wall of the antrum in the vestibule of the omental bursa, (2) the mid-lesser curvature, which are invaded from the metastasis of lesser omentum, and (3) the upper greater curvature by the invasion from splenic hilar metastasis. Region 4 is named as superior omental recessus, which is frequently involved in pseudomyxoma peritonei.

Except for gastric cancer, gastrectomy may be sometimes indicated in patients with peritoneal carcinomatosis from PMP, colorectal cancer, ovarian cancer, and mesothelioma [6, 7]. The reason of gastrectomy is the tumor invasion into the gastric wall. The parts of gastric wall liable to involvement by the disease process are (1) the posterior wall of the antrum in the vestibule of the omental bursa, (2) the mid-lesser curvature, which are invaded from the metastasis of lesser omentum, and (3) the upper greater curvature by the invasion from splenic hilar metastasis (Figure 5). In PMP, almost all invasions are limited in the muscle layer of stomach. If the invasion into the stomach wall is less than 5 cm in diameter, a seromuscular resection or a wedge resection of the whole layer of the stomach using stapler techniques is recommended. Surgeons should decide the necessity of the gastrectomy from the arterial supply for the residual stomach, the areas of invasion, and residual part of the stomach. Importantly, the small bowel should be intact for the safe reconstruction either by esophagojejunostomy or gastrojejunostomy.

#### 2.4.5. Perihepatic Peritonectomy

**Figure 6:**
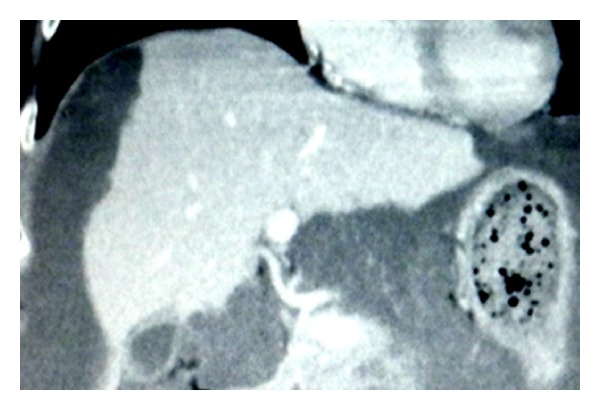
Enhanced CT scan shows tumor located in the hilar, cystic, and umbilical plate, and tumor extended in Glisson's capsule.

**Figure 7:**
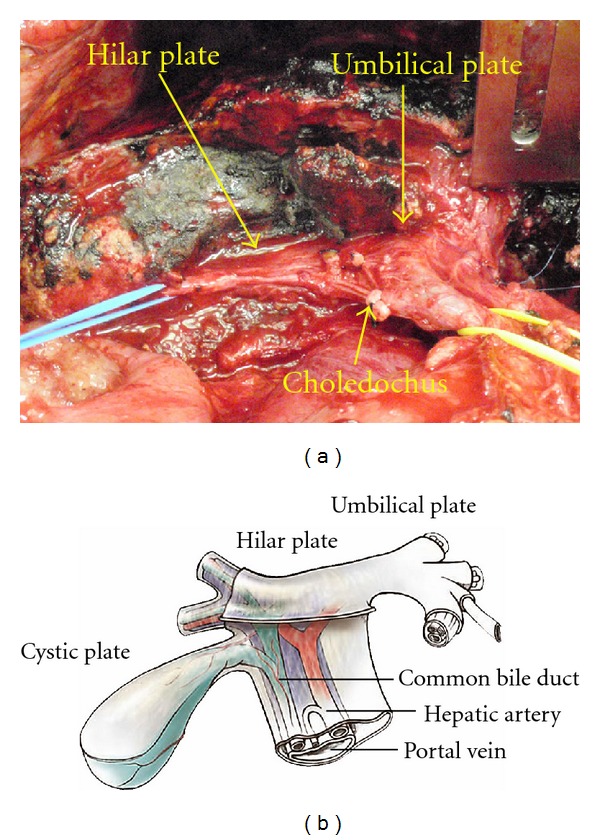
The view of the hilar plate after complete eradication of the infiltrating tumor. The right portal pedicle is tapped by blue tape. The oozing blood from the liver surface is controlled by ABC. Dissection of mucinous tumor from the hilar plate after taping the right portal pedicle branches. This excision only involves surgical removal of Glisson's capsule bearing tumor and approximately 1-2 cm in depth of hepatic parenchyma.

In PC from PMP and mucinous ovarian tumors, hepatoduodenal ligament and liver hilar plate are frequently involved (Figures 6 and 7). The plate system is formed at the level of the liver hilum by coalescence and thickening of Glisson's capsule and vasculobiliary sheaths. This plate system is divided into three parts of connective thickening: the hilar plate that separates the biliary confluence from the inferior part of the quadrate lobe (S4b), the cystic plate that envelops the gallbladder and cystic duct, and the umbilical plate that covers the umbilical portion of the portal vein (Figure 7).

Figure 6 shows the enhanced CT scan showing the tumor located in the hilar, cystic, and umbilical plates and the tumor extended in Glisson's capsule.

The only efficient procedure for hilar metastases is excision, which is followed by complementary treatment. Surgery should start with the dissection of the hepatoduodenal ligament, to identify the limits of the tumor, its mobility, or infiltration of adjacent planes and elements, confirming the existence or absence of infiltration of vascular elements and, in particular, of the portal vein or its branches. Dissection of the hepatic pedicle usually begins with isolation of the artery followed by the biliary tract and portal vein. Aqua dissection enables to identify the second branches of the portal triads (Figure 7). The gall bladder is removed. Lesser omentum is excised routinely. The attachment of the lesser omentum to the caudate lobe and ligamentum venosum is excised and the omental bursa is exposed. The left gastric artery and vein are identified and taped. The lesser omentum is taken all the way from the lesser curvature to the caudate lobe and ligamentum venosum by preserving the left and right gastric vessels.

**Figure 8:**
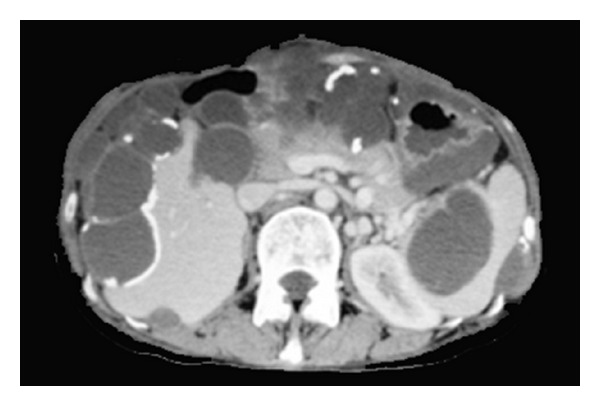
Axial contrast-enhanced CT scan of the upper abdomen demonstrates multiple low attenuated cystic lesions with rim-like calcifications scalloping the liver margin, infiltrating the spleen, and compressing the bowel, pancreas, and left kidney.

**Figure 9:**
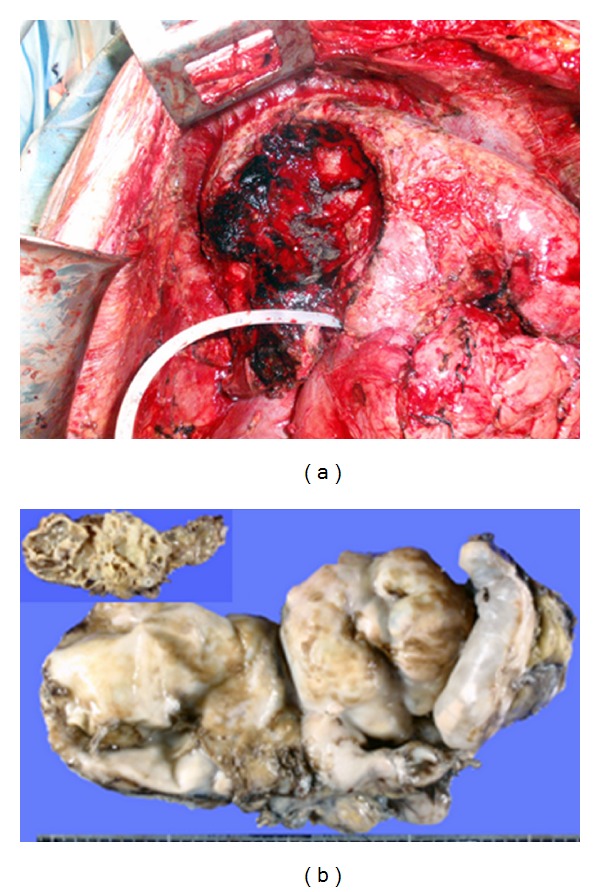
The operative view after enucleating of a large cystic lesion indenting the liver deeply. The resected specimen of the lesion described in Figure 8.

As shown in Figure 8, axial contrast-enhanced CT scan of the upper abdomen demonstrates multiple low attenuated cystic lesions with rim-like calcifications scalloping the liver margin, infiltrating the spleen, and compressing the bowel, pancreas, and left kidney. To remove such lesions, liver capsule near the lesions is cut with electrocautery, and the space between the capsule of the scalloping lesion and liver parenchyma is dissected with scalpel by making a countertraction of tumors.  Figure 9 shows the operative view and resected specimen after enucleating of a large cystic lesion indents the liver deeply.

Peritoneum of the superior omental recess (region 4 in Figure 5), which occupies the caudate lobe, diaphragmatic crus, and anterior wall of vena cava, is removed.

**Figure 10:**
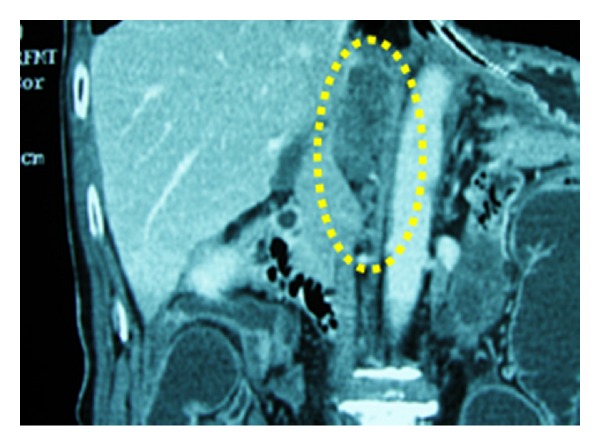
Coronal enhanced CT scan shows tumor located between the inferior vena cava, caudate lobe, and left crus of diaphragm.

Figure 10 shows the tumors in the superior omental recess. By traction of tumors to left side, the capsule of caudate lobe is cut and the tumors with liver capsule and retroperitoneum are peeled off from the caudate lobe, left crural muscle, and vena cava.

**Figure 11:**
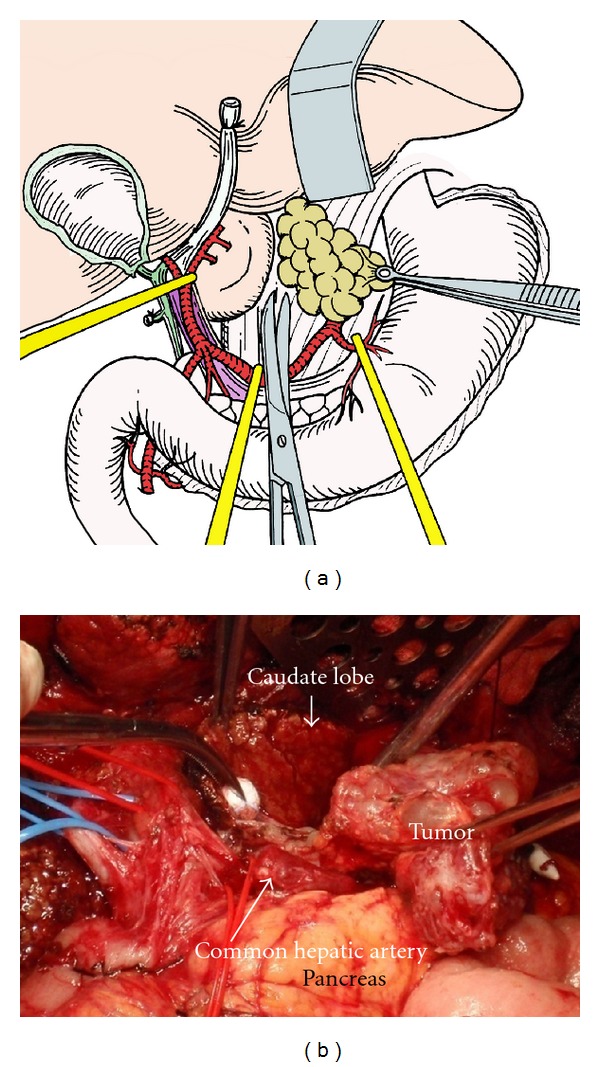
Dissection of the tumor in the superior omental recessus. By traction of tumors to the left side, the capsule of the caudate lobe is cut and the tumors with liver capsule and retroperitoneum are dissected from the caudate lobe, left crural muscle, and vena cave.

**Figure 12:**
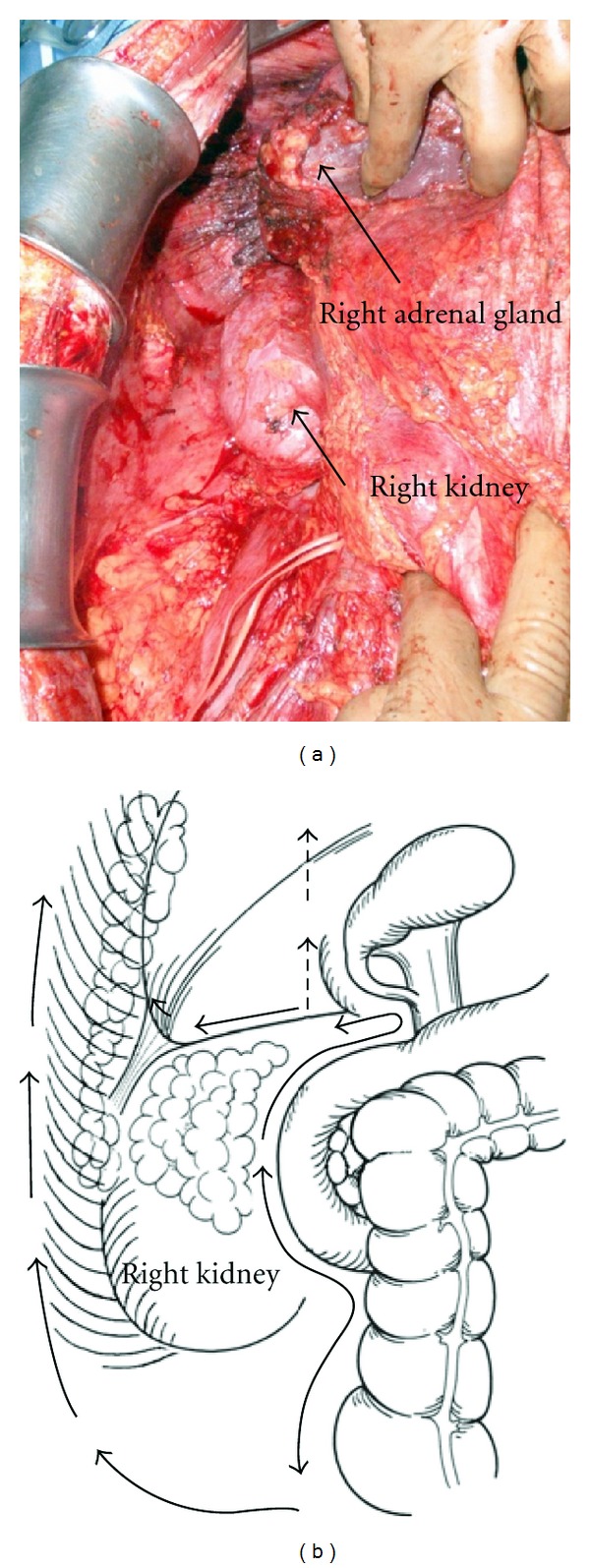
Dissection line of Morrison's pouch. The peritoneum covering Morrisons's pouch is removed with the peritoneum on the right paracolic gutter, right subdiaphragm, and right abdominal wall.

Morrison's pouch and the paracolic gutter are the common sites of tumor implantation. The peritoneum covering Morrison's pouch is removed with the peritoneum on the right paracolic gutter, right subdiaphragm, and right abdominal wall (Figures 11 and 12).

Large tumors attach on the ascending colon and hepatic flexure, and tumors on the paracolic gutter and Morrison's pouch are removed in combination with extended right hemicolectomy.

#### 2.4.6. Pelvic Peritonectomy

The entire pelvic peritoneum is dissected from the anterior inferior abdominal wall, urinary bladder, and retroperitoneum. The peritoneum covering the urinary bladder is dissected, and the rectovesical pouch is completely freed from the urinary bladder and rectum. In males, the space between the seminal vesicle and peritoneum of rectovesical pouch is dissected, lifting the vas deferens off. In females, blood vessels around the uterus are dissected and cut with Ligasure (Valleylab Inc., Boulder, CO, USA). Amputation of the vagina is done at a plane 1 cm below the peritoneal reflection of the Douglass pouch to ensure removal of all tumors occupying the cul-de-sac.

If the tumor invades the anterior rectal wall, the rectum is cut at 1 cm below the peritoneal reflection. Reasonable length of the rectum should be preserved for the anastomosis with the colon or ileum.

#### 2.4.7. Peritonectomy of Small Bowel

The entire small bowel and its mesentery are traced from the duodenojejunal flexure to the ileocecal junction. There are often tumor nodules at paraduodenal recesses covering the ligament of Treitz, and these are easily dissected by the *aqua dissection* technique and resected as well as any other tumor along the way. Then, both sides of the mesentery are inspected and palpated and the tumor nodules excised with electrosurgery.

Complete cytoreduction is aimed by removing all macroscopic tumors by peritonectomy combined with laser or electric fulguration and HIPEC for microscopic PC.

#### 2.4.8. Assessment of Completeness of Cytoreduction

The aim of CRS was to obtain complete macroscopic cytoreduction as a precondition for the application of HIPEC. The residual disease was classified intraoperatively using the completeness of cytoreduction (CC) score [9]. CC-0 indicates no visible residual tumor, CC-1 indicates residual tumor nodules ≤2.5 mm, CC-2 indicates residual tumor nodules between 2.5 mm and 25 mm, and CC-3 indicates residual tumor nodules >25 mm or a confluence of unresectable tumor nodules at any site within the abdomen and the pelvis. CC-2 and CC-3 cytoreductions are regarded incomplete.

### 2.5. Statistical Analyses

All patients were followed up and no patients were lost to follow up. Outcome data were obtained from medical records and patients' interviews. All statistical analyses were performed using the SPSS software statistical computer package version 17 (SPSS Inc., Chicago, USA).

## 3. Results

### 3.1. Completeness of Cytoreduction

CC-0,-1 resections were done in 62/81 (76.5%), 228/420 (54.3%), and 101/166 (60.8%) of patients with colorectal cancer, appendiceal neoplasm, and gastric cancer (Table 1). CC-0,-1 resections of colorectal and appendiceal neoplasm patients with PCI ≤ 20 were performed in 89.4% (59/66) and 86.2% (168/195), but that in gastric cancer patients was done only in 67.6% (100/148). In contrast, 5.6% of gastric cancer patients with PCI ≥ 20 underwent CC-0,-1 resections (1/18), but CC-0,-1 resections in colorectal and appendiceal neoplasm patients were performed in 20.0% (3/15) and 26.6% (60/165), respectively. One gastric cancer patient underwent CC-0 resection for PCI score of 32 which was mucinous adenocarcinoma.

The reasons of CC-2,-3 resections are listed in Table 2. The most frequent reasons were involvement of all peritoneal regions (*N* = 89) and diffuse involvement of small bowel serosa or mesentery (*N* = 113). In appendiceal neoplasms, massive bleeding more than 5 L was the reason to stop operation (*N* = 10). Old age (*N* = 6) and comorbidities (*N* = 4) are also the reasons of CC-2,-3 resections. In appendiceal neoplasms, 6 patients with massive scalloping to the liver hilum or parenchyma showed the reason of CC-2,-3 resections. In gastric cancers, local invasion to the surrounding organs from the primary tumor, positive surgical margin at the esophageal or duodenal stump, and distant lymph node metastasis were found in 6, 3, and 3 patients.

Regarding the correlation between PCI scores of small bowel (SB-PCI) and CC scores in colorectal cancer patients, CC-0,-1 resection was done in 36 of 38 (95%) patients with SB-PCI ≤ 3, but only in 12 of 24 (50%) patients with SBPCI ≥ 4.

In gastric cancer patients, 65 of 78 (83%) of patients with SB-PCI ≤ 3 and 43/83 (52%) of those with SBPCI ≥ 4 underwent CC-0,-1 resections.

In PMP patients, CC-0,-1 resection rate was significantly higher in patients with SB-PCI ≤ 6 (209/265, 79%) than that in those with SB-PCI ≥ 7 (19/155, 12%).

Before December, 2007 (first 3 years), CC-0,-1 resection was done in 82 (42%) of 197 patients. After January, 2008 (next 3 years), when the aqua dissection method was introduced, it was done in 302 (64.3%) of 470 patients, and there was a significant difference (*P* < 0.001). In the first 3 years, complete cytoreduction was done in 14% (11/67) of patients with PCI ≥ 29, but was done in 23.6% (29/122) in the last 3 years. 

### 3.2. Postoperative Mortality and Morbidity

A total of 41 (6.1%) among 667 patients died postoperatively. Mortality rate (3.6%, 14/391) after CC-0,-1 resections was significantly lower than that (8.7%, 24/276) after CC-1,-2 resections (Table 3). Causes of deaths were septic shock (*N* = 14), fistula and peritonitis (*N* = 12), multiple organ failure (*N* = 4), tumor progression (*N* = 4), lung embolism (*N* = 2), cardiac arrhythmia (*N* = 2), bleeding from duodenal ulcer (*N* = 1), and massive abdominal bleeding (*N* = 1). There was no difference between the complication rates and disease categories.

Major complication (grade 3-4 complications) occurred in 126 patients (18.9%). A reoperation was necessary in 36 patients (5.4%). The experienced complications were abdominal abscess (*N* = 45), bowel fistula (*N* = 19), anastomosis or stump leakage (*N* = 18), ileus (*N* = 9), leakage from urinary bladder (*N* = 8), perforation of stomach (*N* = 6), abdominal bleeding (*N* = 5), bile leak (*N* = 3), perforation of diaphragm (*N* = 3), respiratory failure (*N* = 3), renal failure (*N* = 1), arrhythmia (*N* = 1), bleeding from duodenal ulcer (*N* = 1), and others (*N* = 4).

### 3.3. Survival after CRS

 The overall 1-year, 3-year, and 5-year survival rates and median survivals of the three groups are shown in Table 4. Univariate analysis showed that the lymph node status, tumor differentiation, gender, and performance status did not have prognostic impact on survival. The 5-year survival rates after CC-0,-1 resection for colorectal cancer, appendiceal neoplasm, and gastric cancer patients were 28%, 84%, and 17%, respectively. In contrast, those in CC-2,-3 groups were 0%, 50%, and 2% respectively.

The 5-year survival rate of colorectal cancer patients with PCI score ≤ 10 was significantly better than that with PCI score ≥ 11 (*P* < 0.001). In appendiceal cancer, patients with PCI score ≤ 28 survived significantly better than those with PCI score ≥ 29 (*P* < 0.001). Five-year survival rate of gastric cancer patients with PCI score ≤ 6 was significantly better than that of patients with PCI score ≥ 7 (Table 4).

By the multivariate analysis, PCI scores were capable of serving as thresholds for favorable versus poor prognosis in each group and CC scores demonstrated as the significant independent prognostic factors after CRS. In colorectal cancer patients, CC score (CC-0,1 versus CC-2,-3) and PCI score (PCI ≤ 10 versus PCI ≥ 11) emerged as the independent prognostic factors (*P* = 0.031, *P* = 0.0016). RR of patients with CC-2,3 versus CC-0,-1 was 4.63, and that of patients with PCI ≥ 11 versus those with PCI ≤ 10 was 9.98. In gastric cancer patients, CC score (CC-0,-1 versus CC-2,3) was an only independent prognostic factor (*P* < 0.05, *X*2 = 68.47, RR = 26.5).

## 4. Discussion

Current surgical management of the PC can be performed with curative intent and potential long-term survival when a strategy of CRS combined with HIPEC is used to select patients.

Adequate patient selection and the improvement of surgical skills of surgeons are crucial to obtain a complete macroscopic cytoreduction, which is a leading predictor of patient outcome. Adequate patient selection is sometimes difficult for surgeons with experience of small number of cases with PC. Many criteria have to be assessed in each patient: performance status, response to chemotherapies, existence of lymph node and/or hematogenous metastasis, histologic grading, PCI, and comorbidities. Patients with poor performance status, severe comorbidities, and PC already spread to the entire peritoneal cavity are not indicated for complete cytoreduction. In gastric cancer, response after neoadjuvant chemotherapy is one of the selection criteria for CRS [5].

In the surgical treatment of patients with PC from colorectal cancer, appendiceal neoplasm, and gastric cancer, complete cytoreduction is believed as an essential factor for a good prognosis. In the present data, CC-0,-1 and the PCI scores of less than the threshold values for each disease clearly were demonstrated as independent prognostic factors after CRS plus perioperative chemotherapy. Peritonectomy techniques improved the incidence of complete cytoreduction, as compared with the ordinary surgical techniques [6, 9–12]. However, patients often with advanced metastases and a limited life span undergo a comprehensive therapy of multivisceral resection with several intestinal anastomosis and HIPEC. Surgeons are required to safely perform complete macroscopic tumor resection extended across several surgical disciplines, including general surgery, hepatobiliary surgery, urology, and gynecology. The incidences of complete cytoreduction for colorectal cancer, appendiceal neoplasm and gastric cancer in the present paper were 76.5%, 54.3% and 60.8%, respectively. The values appeared reasonable as compared with the results of other big centers [1, 4, 11, 13]. The incidences of complete cytoreduction for colorectal cancer, appendiceal neoplasm, and gastric cancer were reported as 49% ~ 54% (50/102, 271/506) [1, 11], 73.6% (577/783) [4], and 53% (85/159) [13], respectively. The incidences of complete cytoreduction are mainly depending on the surgeons' experiences. A learning curve had been already reported by several authors [12, 14, 15]. Moran et al. reported a decreased mortality rate from 18 to 3% [12] and the Netherlands Cancer Center from 8 to 4% [15]. The present results also demonstrated the the importance of the learning curve and the introduction of new technique of aqua dissection method. The deep invasion or scalloping into the liver hilum and superior omental recess and diffuse small bowel involvement are the limiting factors to achieve complete cytoreduction. 

We developed an aqua dissection technique to guide surgeons to perform a safe tumor dissection through the correct dissection plane, to avoid injury of the important vessels, and to reduce the blood loss. Using this technique, the dissection around the hepatic hilar plate and lateral dissection of the pelvic spaces can be done in a safer and easier manner.

Diffuse small bowel involvement is the most frequent cause of incomplete cytoreduction. Tumor nodules from colorectal and gastric cancer often invade the mesentery where the blood vessels enter the small bowel and this can be especially problematic to resect the tumor nodules without full-thickness injury to the bowel. Once the small bowel is inspected completely, a decision is made to perform resections while leaving adequate bowel length for normal nutritional function and minimizing the number of anastomoses. In colorectal and gastric cancer, the present data demonstrated that the complete cytoreduction rate in patients with SB-PCI ≤ 3 was significantly higher (85.6%, 15/118) than that in patients with SB-PCI ≤ 4 (32.3%, 20/42). In appendiceal neoplasm patients, CC-0,-1 resection rate was significantly higher in patients with SB-PCI ≤ 6 (209/265, 79%) than that in those with SB-PCI ≥ 7 (19/155, 12%). Accordingly, the SB-PCI thresholds for the complete cytoreduction were ≥3 for colorectal and gastric cancer and ≥6 for appendiceal neoplasm. Esquivel et al. reported that there is no surgical option to remove all affected sites of small bowel even if there is evidence of intestinal obstruction at more than one site [16]. In colorectal and gastric cancer, an extended removal of the small bowel will cause not only a short bowel syndrome but also a recurrence within short time. Accordingly, the indication for the extensive bowel resection in colorectal and gastric cancer is limited. On the contrary, Bao and Bartlett reported that 200 cm of small bowel should be maintained in appendiceal neoplasm. These results indicate that the extended resection of small bowel is indicated for the PC from the tumors with a less aggressive biological behavior, like appendiceal neoplasms.

PCI score demonstrated its significant influence on survival, and a PCI score capable of serving as a threshold for favorable versus poor prognosis has been reported. In colorectal cancer, the survival results were significantly better when the PCI was lower than 16 [17, 18]. Sugarbaker also reported a 5-year survival rate of 50% when the PCI was less than 10, a rate of 20% for an index of 11–20, and a rate of 0% for an index >20 [6]. In gastric cancer, Glehen et al. reported that no patients were alive even after complete cytoreduction when the PCI was more than 12. Accordingly, PCI of more than 12 should be contraindicated for CRS and HIPEC [13]. The present study demonstrated that the survival of gastric cancer patients with a PCI ≤ 6 was significantly better than those with a PCI ≥ 7. In contrast, in appendiceal neoplasm, patients with PCI score ≤ 28 showed significant better survival than those with PCI score ≥ 29. Sugarbaker reported that the PCI threshold for appendiceal neoplasm was 20 [4]. Gastric cancer and colorectal cancer have a more aggressive biological behavior than appendiceal neoplasm, and patients with PCI larger than the threshold values should be treated with palliative intent of CRS combined with systemic chemotherapy.

Recently, neoadjuvant intraperitoneal/systemic chemotherapy improves survival results in gastric cancer. Patients who progress or develop extra-abdominal metastases during neoadjuvant chemotherapy may be excluded from an aggressive CRS [5]. In addition, intraperitoneal chemotherapy for gastric cancer is effective to eradicate peritoneal-free cancer cells and small PC nodules [5]. After IP chemotherapy, complete disappearance of cancer cells of PC was observed in 50% of 30 patients with gastric cancer, and also stage migration from stage 4 to stage 1, 2, or 3 was experienced in 33% of patients [5]. These results may indicate that the neoadjuvant chemotherapy can increase the incidence of complete cytoreduction by eradicating PC nodules before surgery. In particular, patients with small bowel involvement should be treated with this strategy before CRS and HIPEC.

## Figures and Tables

**Table 1:** Correlation of CC scores and PCI scores.

	PCI ≤ 10	11 ≤ PCI ≤ 20	21 ≤ PCI ≤ 30	PCI ≥ 31	Total
colorectal cancer					
CC-0,-1	43 (95.5%)	16 (76.2%)	3 (30.0%)	0 (0.0%)	62 (76.5%)
CC-2,-3	2	5	7	5	19
Appendiceal neoplasm					
CC-0,-1	111 (97.4%)	57 (0.3%)	39 (36.4%)	21 (17.8%)	228 (54.3%)
CC-2,-3	3	24	68	97	192
Gastric cancer					
CC-0,-1	95 (79.2%)	5 (21.7%)	0 (0.0%)	1 (25.0%)	101 (60.8%)
CC-2,-3	25	23	14	3	66

**Table 2:** The causes of CC-2,-3 resections.

	Colorectal cancer	Appendiceal neoplasm	Gastric cancer
Involvement of all peritoneal regions	7	71 (22 + old age)	11
Diffuse small bowel involvement	5 (2 + LB^#^, 1 + PH^$^)	86 (15 + LB, 3 + PH,1 + ST^&^)	22 (1 + LG)
Bleeding	0	10	0
Old age	1	5	0
Comorbidity	0	4	0
Positive histologic margin	0	0	6
Local invasion	0	2	3
Lymph node metastasis	0	0	3
Perihepatic involvement	0	6	0
Emergency	1	2	0
others	1	4	1

^#^LB: large bowel involvement, ^$^PH: perihepatic involvement, and ^&^ST: stomach involvement.

**Table 3:** Postoperative mortality and morbidity after cytoreductive surgery.

	No complication	Grades 1-2	Grade 3	Reoperation	Hospital deaths
Colorectal cancer					
CC-0,-1	35 (56.5%)	19 (30.1%)	3 (4.8%)	3 (4.8%)	2 (3.2%)
CC-2,-3	10 (52.6%)	3 (15.8%)	4 (21.0%)	0	2 (10.6%)
Appendiceal neoplasm					
CC-0,-1	128 (56.1%)	44 (19.3%)	32 (14.0%)	19 (8.3%)	5 (2.2%)
CC-2,-3	98 (51.0%)	26 (13.5%)	38 (19.8%)	10 (5.2%)	20 (10.4%)
Gastric cancer					
CC-0,-1	76 (75.2%)	8 (7.9%)	9 (8.9%)	2 (2.0%)	7 (6.9%)
CC-2,-3	45 (68%)	10 (15.2%)	4 (6.1%)	2 (3.0%)	5 (7.6%)

**Table 4:** Survival after CRS in terms of CC score and PCI score.

	Median survivals	1-year survival	3-year survival	5-year survival	Log-rank test (*P*)
	months	(%)	(%)	(%)	*X*2
Colorectal cancer					
CC-0,-1 (*N* = 62)	38.4	93	51	28	*P* = 0.003
CC-2,-3 (*N* = 19)	19.2	33	16	0	*X*2 = 8.53
PCI ≤ 10 (*N* = 45)	NR	95	59	59	*P* < 0.001
PCI ≥ 11(*N* = 36)	18.2	83	37	0	*X*2 = 12.90
Appendiceal neoplasm					
CC-0,-1 (*N* = 228)	NR	99	93	84	*P* < 0.001
CC-2,-3 (*N* = 192)	NR	91	69	50	*X*2 = 41.48
PCI ≤ 28 (*N* = 257)	NR	99	89	76	*P* < 0.001
PCI ≥ 29 (*N* = 163)	49.2	87	63	55	*X*2 = 38.81
Gastric cancer					
CC-0,-1 (*N* = 101)	21.5	70	26	17	*P* < 0.001
CC-2,-3 (*N* = 65)	13.6	59	8	2	*X*2 = 14.90
PCI ≤ 6 (*N* = 111)	21.5	76	23	14	*P* < 0.001
PCI ≥ 7 (*N* = 55)	13.6	57	21	3	*X*2 = 10.48
